# A functional betalain-producing dioxygenase in *Neolecta irregularis* reveals an expanded evolutionary origin of the pathway

**DOI:** 10.3897/imafungus.17.184549

**Published:** 2026-06-05

**Authors:** Pedro Martínez-Rodríguez, M. Alejandra Guerrero-Rubio, Paula Henarejos-Escudero, Samanta Hernández-García, Fernando Gandía-Herrero

**Affiliations:** 1 Departamento de Bioquímica y Biología Molecular A, Unidad Docente de Biología, Facultad de Veterinaria, Regional Campus of International Excellence "Campus Mare Nostrum", Universidad de Murcia, Campus de Espinardo, 30100, Murcia, Spain Regional Campus of International Excellence "Campus Mare Nostrum", Universidad de Murcia Murcia Spain; 2 Universidad Europea de Valencia, Faculty of Health Sciences, Universidad Europea de Valencia, Paseo de la Alameda 7, 46010, Valencia, Spain Faculty of Health Sciences, Universidad Europea de Valencia Valencia Spain; 3 Current Address: Molecular Recognition and Encapsulation Research Group (REM), Health Sciences Department, Universidad Católica de Murcia (UCAM), Campus de los Jerónimos 135, 30107 Guadalupe, Spain Health Sciences Department, Universidad Católica de Murcia (UCAM) Guadalupe Spain

**Keywords:** 4, 5-DOPA-extradiol-dioxygenase, ancient fungus, basal ascomycete, betalain, yellow pigment, *Neolecta irregularis*

## Abstract

Betalains are nitrogen-containing pigments with important biological, ecological, and biotechnological roles, yet their biosynthesis has been described almost exclusively in plants of the order *Caryophyllales* and in a few fungal and bacterial lineages. The early-diverging ascomycete *Neolecta irregularis*, considered a morphological and genomic “living fossil”, offers a unique opportunity to explore the evolutionary origins of this pathway. Here, we combine pigment analysis, gene expression profiling, and biochemical characterization to investigate the betalain-forming potential of this species. Although no betalains were detected in fruiting bodies—whose yellow coloration was instead attributed to riboflavin—RNA-seq data revealed expression of a putative 4,5-DOPA-extradiol-dioxygenase (DODA), the key enzyme in the formation of betalamic acid. We cloned, expressed, and purified the *N. irregularis* dioxygenase (NiDODA) in *Escherichia coli*, demonstrating that it is a monomeric, iron-dependent enzyme capable of producing betalamic acid, muscaflavin, and dopaxanthin from L-DOPA. NiDODA displayed the highest affinity for L-DOPA reported for any DODA to date, and exhibited both 4,5- and 2,3-extradiol-dioxygenase activities. Phylogenetic analysis positioned NiDODA within a distinct fungal branch, expanding the diversity of known betalain-producing enzymes.

## Introduction

Betalains are water-soluble nitrogenous compounds that constitute the main pigment in most species of the order *Caryophyllales*. Interest in the study of these molecules lies not only in their role as a taxonomic marker (Brockington et al. 2011) but also in their known health-promoting properties (Martínez-Rodríguez et al. 2022). These phytochemicals exhibit a wide range of bioactive effects, among which their antioxidant, anti-inflammatory, chemopreventive, and neuroprotective capacities stand out (Martínez-Rodríguez et al. 2022). As a result, the search for novel natural sources of betalains has been intensified, and new biotechnological protocols have been developed to increase production yield (Guerrero-Rubio et al. 2019).

Betalains provide color ranging from yellow-orange to red-violet in the organisms that produce them. In nature, there are two main types of betalains: yellow betaxanthins and violet betacyanins. Both types of pigments share betalamic acid as their structural and functional unit (Carreón-Hidalgo et al. 2022). The biosynthesis of this molecule involves the enzyme 4,5-DOPA-extradiol-dioxygenase, which uses L-DOPA as substrate to produce the unstable intermediate 4,5-seco-DOPA, which spontaneously gives rise to betalamic acid (Khan and Giridhar 2015). Betaxanthins result from the spontaneous condensation of betalamic acid with amines or amino acids, while betacyanins are formed through the conjugation with indole-type derivatives such as *cyclo*-DOPA.

In addition to the plants, betalains have also been found in fungal species such as *Amanita*, *Hygrocybe*, and *Hygrophorus* (Belhadj Slimen et al. 2017). Likewise, the ability to synthesize these pigments has been described in bacteria such as *Gluconacetobacter diazotrophicus* (Contreras-Llano et al. 2019) and *Escherichia coli* (Gandía-Herrero and García-Carmona 2014), as well as in the cyanobacteria *Anabaena cylindrica* (Guerrero-Rubio et al. 2020), and more recently also in animals, such as water bears (*Hypsibius henanensis*) (Li et al. 2024). This work aims to expand the understanding of betalain-producing capacity in alternative species. For this purpose, the research focuses on the unique organism *Neolecta irregularis*. This fungus, belonging to the Subphylum *Taphrinomycotina*, occupies a basal position within the Phylum *Ascomycota*, making it a species of particular evolutionary interest (Nguyen et al. 2017). *N. irregularis* exhibits simple, fleshy, bright yellow fruiting bodies with tongue-like shapes emerging from the soil, which has led to their description as “earth tongues” (Schoch et al. 2009). Unlike other *Ascomycota* species, *Neolecta* retains traits considered archaic, both morphologically and genetically, placing it in an intermediate position between filamentous fungi and yeasts (Landvik et al. 2001). Beyond its unusual morphology, *Neolecta irregularis* holds exceptional evolutionary significance. As one of the very few surviving members of the *Taphrinomycotina* and the only genus within the *Neolectomycetes*, *Neolecta* occupies a uniquely basal position in the *Ascomycota* phylogeny, retaining genomic and developmental features considered ancestral within the phylum. Its combination of yeast-like characteristics with simple, fruiting-body–forming structures has long been regarded as an evolutionary enigma, raising questions about how early fungal lineages organized multicellularity, metabolism, and ecological interactions. The rarity of *Neolecta* specimens and the inability to cultivate the organism have further limited functional studies, leaving major aspects of its physiology unresolved. Consequently, investigating its metabolic capacities, particularly those involving specialized or lineage-specific pathways, offers an unusual window into early fungal evolution and the diversification of metabolic innovations.

Its singular phylogenetic position raises a broader unresolved question in evolutionary biochemistry: why do non-plant organisms retain genes encoding enzymes traditionally associated with betalain biosynthesis? The occurrence of betalain-forming DOPA-dioxygenases in fungi, bacteria, cyanobacteria, and even animals suggests that their distribution may not be an isolated curiosity but the consequence of deeper evolutionary processes. Whether these enzymes represent ancestral metabolic capacities that precede the diversification of major eukaryotic lineages or instead reflect independent acquisitions through horizontal gene transfer or by convergent evolution, remains an open and largely unexplored problem. Addressing this question is essential for understanding how pigment-related metabolic pathways emerge, persist, or disappear across distant branches of the tree of life. In this regard, the present work analyzes the betalain pigment-forming capacity of *Neolecta irregularis* species by transcriptomic analysis and characterizing its 4,5-DOPA-extradiol-dioxygenase (NiDODA) enzymatic activity. The cloning, expression, purification, and molecular and functional characterization of NiDODA increase the limited number of recognized dioxygenases capable of producing betalamic acid, being the second fungal dioxygenase characterized to date. The identification of this pigment-producing capability in such a distinctive lineage expands the known phylogenetic scope of betalains, highlighting that key metabolic innovations associated with betalains may have deeper and more ancient origins than previously appreciated.

## Methods

### Reagents, chemical products, and cells

Kanamycin (Km), chloramphenicol (Cm), isopropyl-β-D-1-thiogalactopyranoside (IPTG), 3,4-dihydroxy-L-phenylalanine (L-DOPA), trifluoroacetic acid (TFA), and sodium ascorbate were obtained from Merck KGaA (Darmstadt, Germany). Water and HPLC-grade acetonitrile were obtained from Fisher Scientific (Loughborough, UK). *E. coli* BL21 (DE3) pLysS cells were obtained from Invitrogen (Waltham, MA, USA). HIS-Select Cobalt Affinity Gel was obtained from Merck KGaA. All other chemicals and reagents were obtained from Merck KGaA.

### Fungal material and pigment extraction

Two independent specimens of *N. irregularis* were obtained from Denver Botanic Gardens fungarium (DBG; Denver, CO, 80206) under catalog numbers DBG-F-033461 and DBG-F-031510. The fungal material was stored at 4 °C upon receipt. The material used for pigment extraction was the yellow tongues characteristic of *N. irregularis* specimens. The tongues were placed in an Eppendorf tube, and the pigments were extracted by crushing using a mini-mortar at 4 °C in 10 mM sodium phosphate buffer, pH 6.0, supplemented with 15 mM sodium ascorbate. The volume of buffer added was 250 μL per 0.02 g of fungi sample (wet weight). The extracts were then centrifuged twice at 10,000 rpm for 5 min to remove fungal debris and stored at -20 °C until further analysis.

### Macroscopy images and fluorescence imaging

Macroscopy images were obtained using a Leica Z6 APO macroscope with incident light beam (Leica Microsystems, Wetzlar, Germany) attached to a digital camera (Leica DC500). For fluorescence imaging, the filter cube I3 (Leica Microsystems) was used, the excitation band-pass filter limited the excitation wavelength to the range 450–490 nm, while the long-pass emission filter captured the fluorescence emitted by the sample at wavelengths longer than 510–515 nm.

### RNA extraction and RNA-seq analysis

Total RNA extraction was performed with TRIzol® following the protocol of PureLink^TM^ RNA Mini Kit from Invitrogen (Carlsbad, CA, USA). RNA integrity was evaluated using an Agilent 2100 Bioanalyzer (Agilent Technologies) with the 2100 Expert Eukaryote Total RNA Nano chip, prior to library construction. cDNA libraries were prepared using the Illumina Stranded mRNA Prep, Ligation kit (Illumina, catalog no 20040534), following the manufacturer’s instructions. The kit performs a PolyA capture followed by a ligation-based addition of adapters to obtain the libraries. Libraries were quantified with the Qubit 4 Fluorometer (Thermo Fisher Scientific) using the dsDNA High Sensitivity Assay. Fragment size distribution and library quality were further assessed using the Agilent 2100 Bioanalyzer with the dsDNA High Sensitivity kit (Agilent Technologies). Library preparation and transcriptome sequencing were carried out at the Molecular Biology Service (ACTI, University of Murcia, Spain) using the NextSeq 1000/2000 system and P3 flow cell chemistry (200 cycles), generating 75 bp paired-end reads with a minimum of 25 million reads per sample.

### Bioinformatic analysis

Preprocessing of the Illumina RNA-seq raw reads was done using fastp (v0.20.0) (Chen et al. 2018) to remove low-quality sequences. Quality of the reads before and after the fastp processing was estimated by FastQC v0.11.9 (http://bioinformatics.bbsrc.ac.uk/projects/fastqc). Then, clean reads were mapped to the *N. irregularis* DAH-1 v1.0 reference genome (https://mycocosm.jgi.doe.gov/Neoirr1) using hisat2 v4.8.2 (Kim et al. 2015), and Samtools v0.1.19 was used to translate SAM file to BAM file and then to sort BAM files using default parameters (Li et al. 2009). FeatureCounts v2.0.1 in the subread package was used to generate read counts for each sample (Liao et al. 2013). The detection and removal of low-count features across samples was performed by using the NOISeq R package (Tarazona et al. 2011), and the expression counts normalization was performed by using the reads per kilobase of transcripts per million (RPKM) method.

### *N. irregularis* DODA sequence and cloning

The gene sequence of the NiDODA, deposited at the National Center for Biotechnology Information (NCBI, Bethesda, MD, USA) under ID code OLL26206.1 (Nguyen et al. 2017), was used as a template to obtain a codon-optimized sequence for expression in *E. coli* (Biomatik, Cambridge, Ontario, Canada). The genome of the fungus *N. irregularis*, accessible through NCBI under accession number LXFE01000000 (BioProjectID 167926) (Nguyen et al. 2017) was identified to contain a putative 4,5-DOPA-extradiol-dioxygenase protein deposited under GenBank accession number LXFE01000220.1 and under GenPept accession number OLL26206.1 by analysis screening with tblastn tool (Camacho et al. 2009) using the *G. diazotrophicus* 4,5-DODA sequence (Contreras-Llano et al. 2019) as template.

The synthetic DODA gene, an 813-bp product, was expressed in the recombinant plasmid pET-28a. This plasmid encodes an additional sequence at the C-terminal end containing a 6xHis tag. Subsequently, thermocompetent *E. coli* BL21 (DE3) pLysS cells were employed to express the new pET-28a-NiDODA. The transformed cells were seeded on LB agar plates containing Cm (34 μg mL^–1^) and Km (100 μg mL^–1^) and placed at 37 °C overnight. The formation of cell colonies on the plate denoted the correct transformation of the competent cells.

### Expression and purification of NiDODA

Transformed pET-28a-NiDODA cells were grown in LB medium supplemented with Cm (34 μg mL^–1^) and Km (100 μg mL^–1^) at 37 °C under continuous orbital shaking until an optical density at 600 nm (O.D. _600_) of 0.8–1.2 was reached. Expression of the DODA protein was optimized at two temperatures (20 and 25 °C) by adding different concentrations of IPTG (0.1, 0.5, and 1 mM) used as expression inducer. Once the optimal conditions were determined, protein production was scaled up to 1 L. After the induction, the temperature was lowered to 20 °C, and the cells were left under orbital shaking for 20 h to allow protein expression. Then the cells were centrifuged at 6,000 g for 10 min at 4 °C, resuspended in Milli-Q water, and centrifuged again, thus washing out the LB surplus with the second centrifugation. The cell pellet was then resuspended in 50 mM sodium phosphate buffer, pH 8.0, containing 0.3 M NaCl and 50 μM FeCl_2_, and disrupted using a Branson digital sonifier (20% amplitude for 50 seconds) (Branson Ultrasonics). The cell lysate was centrifuged at 13,000 g for 10 min, the pellet was discarded, and the NiDODA enzyme present in the supernatant was purified using HIS-Select Cobalt Affinity Gel (Merck KGaA) following the manufacturer’s instructions, with some modifications (50 μM FeCl_2_ and 20 mM imidazole were added to the buffers). The 6xHis tag present in the enzyme allows its selective binding and retention to the cobalt-charged particles of the gel, while the other proteins of the sample were washed away. The bound dioxygenase protein was released using an elution buffer with a higher concentration of imidazole (250 mM) than the binding and washing buffers. Subsequently, the DODA enzyme was desalted using PD10 columns (GE Healthcare, Milwaukee, WI, USA) using 20 mM sodium phosphate buffer, pH 6.8 as elution buffer. Protein quantification was performed using the Bradford protein assay (Bio-Rad Laboratories, Hercules, CA, USA) (Bradford 1976). Finally, the samples were analyzed by sodium dodecyl sulfate polyacrylamide gel electrophoresis (SDS-PAGE) (Matsumoto et al. 2019). Samples were denatured and loaded on 15% polyacrylamide gels, and the gels were run for 45 min at 200 V. Prestained Protein Ladder marker EZ-Run (170–10 KDa) (Thermo Fisher Scientific) was loaded in the first well of the gels to estimate the protein molecular weight. Then the gels were stained using the standard Coomassie blue method (Dong et al. 2011).

### Gel filtration

Pure recombinant protein samples were applied to a Superdex 200 10/300 GL column, which was equilibrated with 50 mM sodium phosphate buffer, pH 7.5, containing 150 mM NaCl. The protein was eluted using the same buffer at a flow rate of 0.5 mL min^–1^. Elutions were performed using an Äkta purifier apparatus (General Electric Healthcare, Chicago, Illinois, USA) and monitored at 280 nm. The following protein markers (Merck KGaA, Darmstadt, Germany) were used for column calibration: cytochrome C (12.4 kDa), carbonic anhydrase (29 kDa), albumin (66 kDa), alcohol dehydrogenase (150 kDa), and β-amylase (200 kDa).

### HPLC-ESI-Q-TOF-MS protein analysis

For the determination of protein molecular mass, the HPLC-electrospray (ESI) Q-TOF-MS technique (Agilent 1290 Infinity II + Q-TOF 6550 with Dual ESI Jet Stream/i-funnel; Agilent Technologies, Santa Clara, CA, USA) was used. The column used was the Supelco Discovery BioWidePore C5, 2.1 × 10 cm and 5 μm particle, specially designed for large biomolecules such as proteins. The flow rate was 0.4 mL min^–1^. The sample was injected onto the column and eluted using an acetonitrile gradient. Before entering the MS, the characteristic signal of the protein was recorded using an absorbance detector at a wavelength of 280 nm. Subsequently, the spectrum of the main peak was subjected to a mathematical deconvolution algorithm to associate different *m/z* peaks with a common molecular mass but different charge states.

### Trypsin digestion

The protein sample was prepared in 100 μL of 50 mM NH_4_HCO_3_ buffer, pH 8.0, to which 0.02% ProteaseMAX surfactant (Promega, Madison, WI, USA) was added. Subsequently, sample reduction was performed using 10 mM dithiothreitol at 56 °C for 20 min, followed by alkylation with 50 mM iodoacetamide at 25 °C for 20 min. One microgram of proteomic grade trypsin (Promega, Madison, WI, USA) was then added, and the sample was incubated for 4 h at 37 °C. To collect the fragmented proteins, centrifugation was performed at 15,000 g for 1 min, and 0.5% trifluoroacetic acid (TFA) was added to stop the digestion. The resulting peptides were subjected to cleanup using C-18 ZipTips (Millipore, Burlington, Massachusetts, USA) and subsequently evaporated using an Eppendorf vacuum concentrator (model 5301).

### ICP-MS

For the experimental determination of the cofactor used by the NiDODA, analyses were performed using Inductively Coupled Plasma Mass Spectrometry (ICP-MS) on an Agilent 7900 instrument. Prior to analysis, samples were digested using an UltraWAVE microwave digestion system (Milestone Srl, Italy). Nebulization was carried out with a Micro Mist nebulizer and a Scott-type double-pass spray chamber (quartz). The quartz torch included an injector with an internal diameter of 2.5 mm. The system operated at a radio frequency of 27.12 MHz and a power output of 1550 W. Argon flow rates were 15 L min^–1^ for plasma, 0.9 L min^–1^ for the auxiliary, and 1.05 L min^–1^ for the nebulizer. The solution uptake rate was 0.9 mL min^–1^. Nickel sampler and skimmer cones with internal diameters of 1.0 mm and 0.45 mm, respectively, were used. The interface vacuum pressure was 1.4 Torr, and the analyzer chamber pressure was 8 × 10^–7^ Torr. Data acquisition was performed in quantitative spectrum mode, with a dwell time of 300 ms, 100 sweeps per replicate, and 3 replicates per sample.

### HPLC analysis

To carry out analytical HPLC separations, a Shimadzu (Kyoto, Japan) LC-20AD apparatus was used, which was equipped with an SPD-M20A photodiode array detector. Reversed-phase chromatography was performed using a 250 × 4.6 mm Kinetex C-18 column with 5 μm particle size (Phenomenex, Torrance, CA, USA). A linear gradient was used for elution of the compounds, using water with 0.05% trifluoroacetic acid (TFA) as solvent A, and acetonitrile with 0.05% TFA as solvent B. This linear gradient was carried out for a total time of 35 min, starting from an initial composition of 0% B and reaching up to 35% B, maintaining a constant temperature of 25 °C. The flow rate used was 1 mL min^–1^, and the sample injection volume was 50 μL.

### Mass spectrometry analysis of metabolites

An Agilent 6220 TOF/Q-TOF-MS spectrometer (Agilent Technologies) with an atmospheric pressure chemical ionization interface (ESI-APCI) was used to perform precise mass determination of metabolites. Samples were ionized in positive mode, applying a capillary voltage of 3.5 kV. For sample drying, nitrogen gas was used at a temperature of 350 °C, with a flow rate of 11 L min^–1^, and a nebulization pressure of 40 psi. All data obtained were processed using Agilent Technologies MassHunter software.

### Absorbance spectroscopy

The enzymatic activity of NiDODA was determined by a continuous spectrophotometric method measuring absorbance at 414 nm, signal associated with the presence of betalamic acid and muscaflavin (Girod and Zryd 1991; Gandía-Herrero and García-Carmona 2012). Unless otherwise indicated, the reaction medium consisted of 50 mM sodium phosphate buffer at pH 6.0, a L-DOPA concentration of 7.6 mM, and 100 mM sodium ascorbate, with a final volume of 300 μL. Measurements were performed at 25 °C in 96-well plates using a Synergy HT plate reader from Bio-Tek Instruments (Winooski, USA). Betalamic acid solutions of known concentration were used to prepare a calibration curve using the same device. For the quantification of betalamic acid, a molar coefficient of 24,000 M^–1^ cm^–1^ was used (Trezzini et al. 1991). Kinetic analysis of the data was performed by fitting a nonlinear regression using SigmaPlot Scientific Graphing software version 14.0 for Windows (2017, Systat Software, San Jose, CA, USA).

### Production of betalains in *E. coli* (pET-28a-NiDODA) cultures

Microbial cultures of *E. coli* BL21 transformed with plasmid pET-28a-NiDODA were grown in LB medium supplemented with Cm (34 μg mL^–1^) and Km (100 μg mL^–1^). The culture was maintained at 37 °C under orbital shaking until it reached an O.D. _600_ of 0.8–1.2. Next, 1 mM IPTG was added to the culture to induce heterologous expression of NiDODA. After that, the culture was maintained at 20 °C for 20 h. Then, the culture was centrifuged at 6,000 g for 10 min at 4 °C. The cell pellet was resuspended in Milli-Q water and centrifuged again, thus washing out the LB surplus. The resulting pellet was then resuspended in Milli-Q water supplemented with 7.6 mM L-DOPA and 15 mM sodium ascorbate. This reaction medium was maintained at a constant temperature of 20 °C with orbital shaking for 120 h. Samples were taken at different times to follow the formation of the reaction products. These samples were centrifuged for 1 min at 7,500 rpm, and the supernatant obtained was analyzed by HPLC.

### Phylogenetic analysis of betalamic acid-forming enzymes

A BLAST search was first performed using the *N. irregularis* coding sequence with accession number ID OLL26206.1 at the National Center for Biotechnology Information (NCBI, Bethesda, MD, USA), to identify homologous sequences. The 10 retrieved sequences with the highest percent identity were selected for subsequent analysis. The phylogenetic relationship of *N. irregularis* with betalamic acid-forming enzymes was inferred by phylogenetic reconstruction based on the protein sequence comparison of DODA homologues (Suppl. material 1: table SS1), including the 10 retrieved sequences from BLAST search. Multiple sequence alignment was performed by using MAFFT (Multiple Alignment using Fast Fourier Transform) program from EMBL-EBI (European Molecular Biology Laboratory – European Bioinformatics Institute) web server and subsequently processed in MEGA12 to generate a maximum-likelihood phylogenetic tree via the Jones-Taylor-Thornton (JTT) model of amino acid substitutions with 1,000 bootstrap replicates (Letunic and Bork 2019; Madeira et al. 2019).

## Results

### Gene expression in *N. irregularis* specimens

RNA-seq analysis was performed with the fungal samples. The summary of the sequence data generated by RNA-seq and genome mapping of *N. irregularis* is shown in Suppl. material 1: table S2. As can be seen, the results obtained are similar for two independent samples from different specimens of *N. irregularis*. For the first time, it has been possible to extract RNA from this species and analyze its gene expression.

The gene expression results were deposited and are freely available in the NCBI Gene Expression Omnibus (GEO) database under the accession GSE300630.

The RNA-seq analysis showed that the samples expressed several enzymes of the betalain biosynthetic pathway, including Cytochrome P450 and the DODA enzyme (Suppl. material 2). However, the enzymes prephenate dehydrogenase (NADP+) and tyrosine aminotransferase, key enzymes in tyrosine production, were not detected. Furthermore, in accordance with the yellow appearance of the specimens, the samples expressed riboflavin synthase—the final enzyme in riboflavin biosynthesis which catalyzes the conversion of two molecules of 6,7-dimethyl-8-ribityllumazine into one molecule of riboflavin. The results also indicated the expression of the enzyme responsible for FMN (flavin mononucleotide) production, and all transcripts can be found in GEO (GSE300630).

### Pigment analysis of fruiting bodies of *N. irregularis*

**Figure 1.**
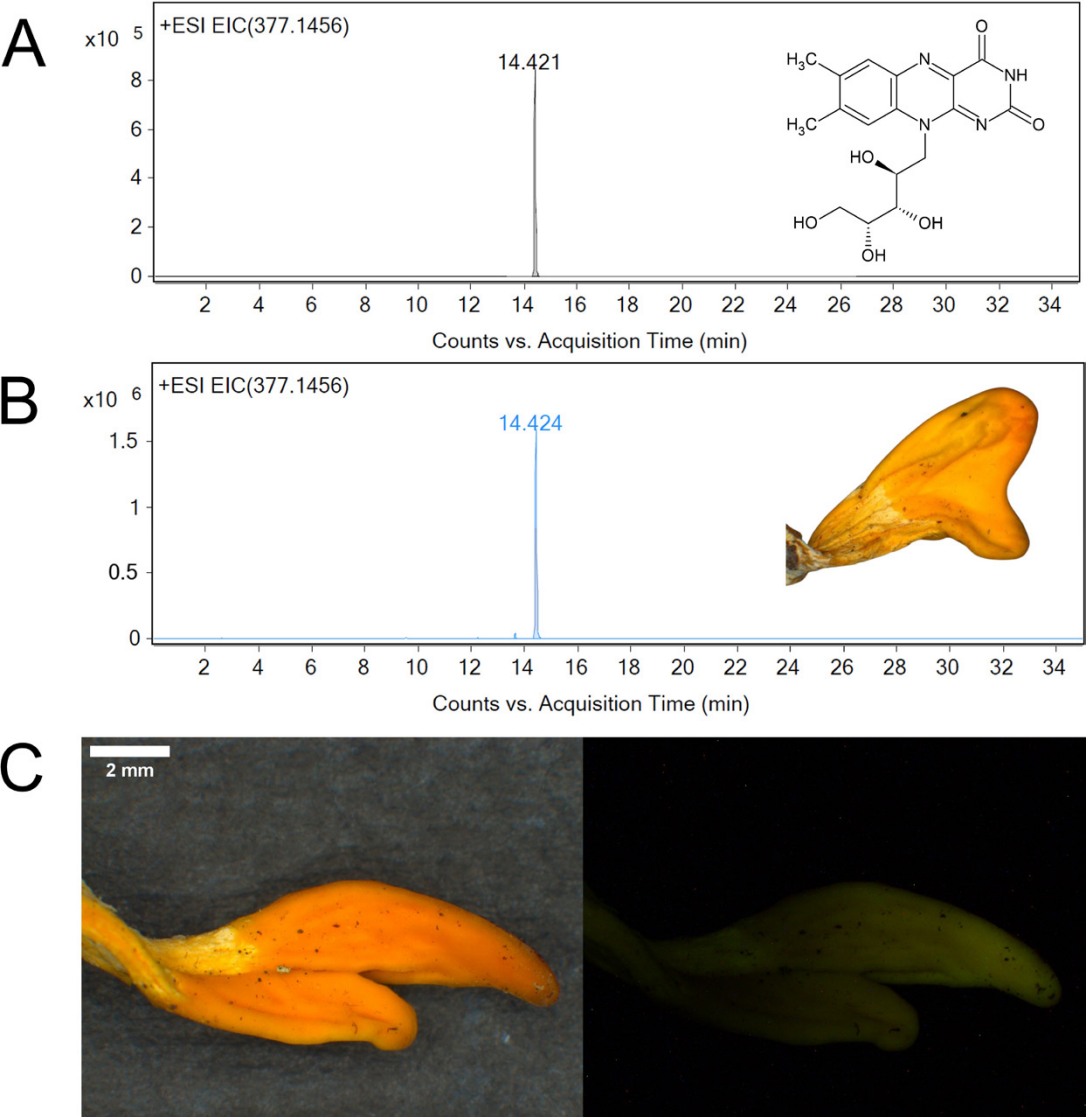
Detection of riboflavin in *N. irregularis*. **A, B**. Chromatograms for the HPLC-ESI-TOF-MS analysis. Standard riboflavin was followed at EIC 377.1456 *m/z* (**A**), and the same peak was detected in *N. irregularis* extracts (**B**). **C**. Macroscopic image of two tongues of *N. irregularis* visualized in bright field (left) and in fluorescence (right) using the I3 fluorescence filter cube. Scale bar: 2 mm.

The pigment profile of *N. irregularis* extracts was analyzed by HPLC-ESI-Q-TOF. As a first approach, we screened the samples for betalains typically reported in fungi such as *Amanita muscaria* (Stintzing and Schliemann 2007), using authentic standards for muscaflavin, several amino-acid-derived betaxanthins (ethanolamine-betaxanthin, alanine-betaxanthin, serine-betaxanthin, muscimol-betaxanthin, valine-betaxanthin, threonine-betaxanthin, leucine-betaxanthin, glutamine-betaxanthin, methionine-betaxanthin, dopamine-betaxanthin (miraxanthin V), histidine-betaxanthin (musca-aurin VII), musca-aurin I, phenylalanine-betaxanthin, dopaxanthin, tryptophan-betaxanthin, and musca-aurin II), dopaxanthin, betalamic acid, and L-DOPA, prepared according to the published protocol (Guerrero-Rubio et al. 2019). However, none of these molecules were detected in the extracts of *N. irregularis*. Instead, the main pigment responsible for the yellow coloration of the fruiting bodies was identified as riboflavin (vitamin B2), as indicated by its retention time (Rt = 14.42 min) and its exact mass (*m/z* 377.1464), which matched the riboflavin standard (*m/z* 377.1456). Fluorescence imaging under blue excitation further supported this assignment, given the characteristic yellow-green fluorescence emitted by riboflavin. Fig. 1C shows the fluorescence displayed by the organism in accordance to light emission by riboflavin (Northrop-Clewes and Thurnham 2012) when excited in the blue light range (380–450 nm).

This unexpected result raises a key biological question: if the fungus possesses - and expresses - the gene encoding a DOPA-dioxygenase (DODA), as revealed in the transcriptomic assays, then why does it not display betalain-derived yellow pigmentation? The absence of detectable betalains in the native tissues, despite the presence of the DODA gene in the genome and its expression in the transcriptome, suggests that *N. irregularis* may lack the necessary substrates *in vivo* or that the pathway is inactive under natural conditions. The analysis in this section reveals the absence of L-DOPA, the primary substrate of the pathway, in the biological samples. Consequently, this finding becomes a starting point since it motivates the subsequent characterization of the DODA enzyme itself to determine whether the fungus retains a functional betalain-forming capacity.

Thus, the pigment analysis not only rules out betalains as contributors to coloration in *N. irregularis* but also frames a central question on the apparent contradiction with the transcriptomic assays: does the encoded DODA enzyme retain biochemical activity capable of producing betalamic acid, and if so, what does this imply about the evolutionary maintenance of this gene in non-plant lineages?

### *N. irregularis* 4,5-DODA sequence, expression, and purification

The genome of the fungus *N. irregularis* is stored in the DDBJ/ENA/GenBank database under accession number LXFE00000000. Additionally, it is accessible through NCBI under accession number LXFE01000000 (BioProjectID 167926) (Nguyen et al. 2017). From the complete genome and using the *G. diazotrophicus* 4,5 DODA sequence (Contreras-Llano et al. 2019) as template, the sequence corresponding to the 4,5-DOPA-extradiol-dioxygenase protein, deposited under GenBank accession number LXFE01000220.1 and under GenPept accession number OLL26206.1 at NCBI, was found.

The OLL26206.1 protein sequence was used as a template to obtain a synthetic sequence optimized for expression in *E. coli* cells. The modifications made during the optimization process did not alter the encoded amino acid sequence. The obtained codon-optimized sequence was inserted into the multiple cloning site of the expression vector pET-28a, resulting in the recombinant plasmid pET-28a-NiDODA. The expression vector includes an LAC promoter, to induce the enzyme expression and a 6xHIS tag that adds six histidine molecules to the expressed protein to enable the purification.

To carry out the heterologous expression of the enzyme, *E. coli* BL21 (DE3) pLysS cells were transformed with the recombinant plasmid pET-28a-NiDODA. IPTG was used as inducible promoter of the enzyme expression. Thus, to secure the maximum expression of the enzyme, an optimization process was performed, including different concentrations of the inducer IPTG at two temperatures. After induction, the substrate L-DOPA 7.6 mM and 15 mM sodium ascorbate was added to the reaction medium. The production of dopaxanthin was measured by HPLC and used as an indicator of the NiDODA expression level. The induction with 1 mM IPTG at 20 °C was the condition that yielded the highest amount of dopaxanthin (Suppl. material 1: fig. S2). Therefore, these conditions were used for the rest of the experiments.

**Figure 2.**
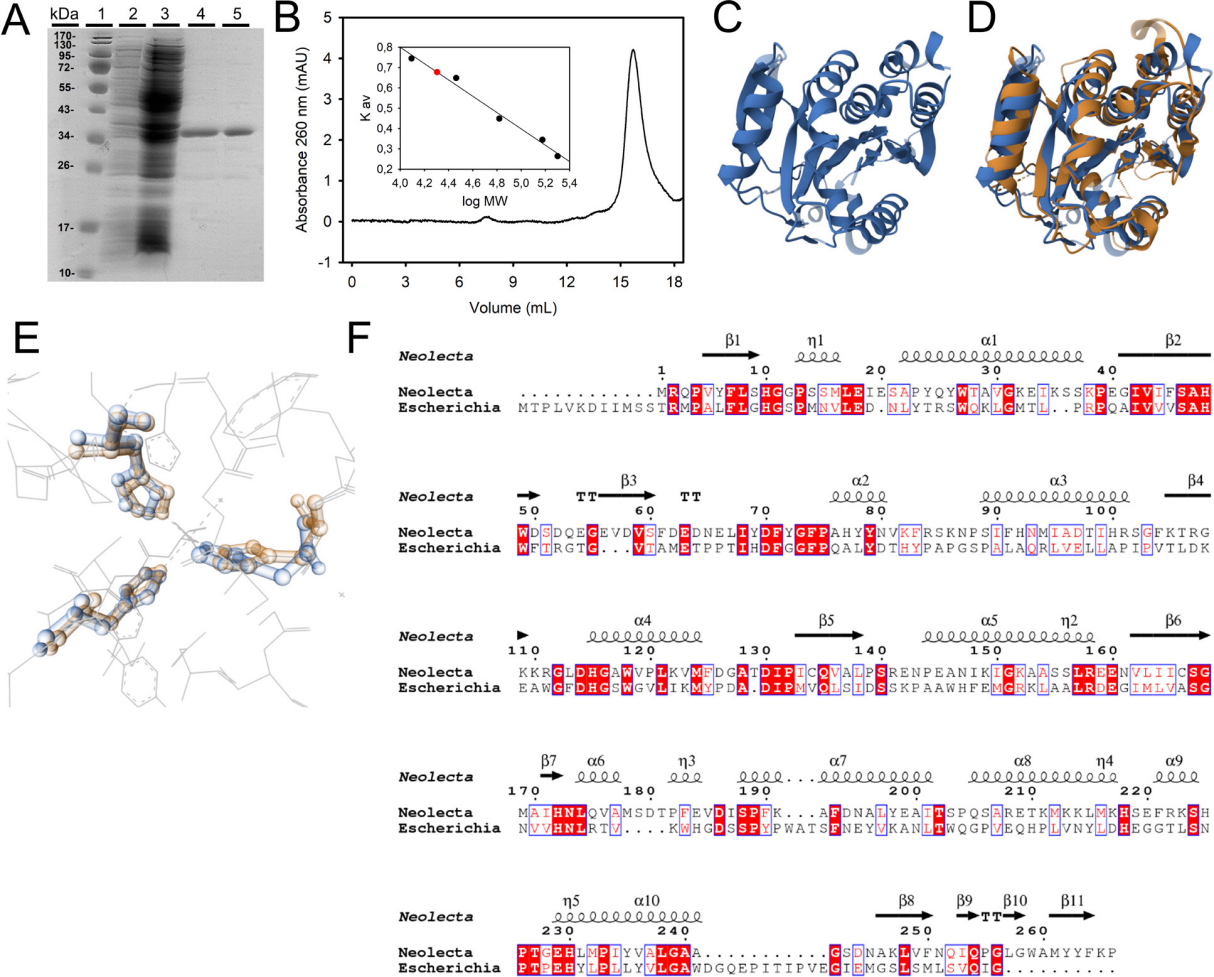
NiDODA structural characterization. **A**. Electrophoretic analysis (SDS-PAGE) of the heterologous expression and purification of the NiDODA expressed in *E. coli* BL21 (DE3) pLysS cells. Lane 1, molecular weight markers; lane 2, soluble protein content of cells harvested before IPTG addition; lane 3, soluble protein content of cells harvested 20 h after induction with IPTG (1 mM); lane 4, eluted protein after affinity chromatography purification; lane 5, final purified protein obtained after desalting using PD-10 columns. **B**. Analysis of the NiDODA by gel filtration chromatography. Elution was monitored at λ = 260 nm. Inset: Calibration with standard proteins. The purified protein is indicated as a red dot on the calibration curve. K_AV_ is an elution volume parameter that characterizes the size of the protein within a fractionating column (K_AV_=(V_e_−V_o_)/(V_t_−V_o_)). **C**. 3D model of the NiDODA. **D**. Overall structural comparison between NiDODA (blue) with the structurally characterized protein YgiD (PDB:2PW6) from *E. coli* (brown). **E**. Close-up view of the three conserved histidine residues involved in the configuration of the active site of NiDODA (His10, His48, and His230, shown in blue) compared to those of *E. coli* (His22, His57, and His234, shown in brown). Residues in the surroundings shown in grey correspond to the crystal structure of YgiD. **F**. Sequence comparison of NiDODA with the structurally characterized protein YgiD (PDB:2PW6) from *E. coli*. Sequence alignment using structural information was performed with Expresso and visualized with ESPript.

NiDODA purification process was evaluated by denaturing SDS-PAGE, revealing that recombinant NiDODA was purified to homogeneity after affinity separation (Fig. 2A). The results of the enzyme purification process are shown in Table 1. The final pure protein was used to carry out its structural and kinetic characterization.

**Table 1.** Expression and purification of *N. irregularis* dioxygenase.

Sample	Volume (mL)	Protein (mg mL^-1^)	Total prot. (mg)	Activity (µM min^-1^)^a^	Specific activity (µmol min^-1^ mg^-1^)	Purif. fold	Yield (%)
Crude extract^b^	7.5	17.73	132.975	0.063	0.011	1.0	100
Co^2+^ resin	3.5	0.495	1.733	0.036	0.218	20.5	26.72

^a^ Activity was determined using 100 μL of protein solution under the assay conditions. ^b^ Crude extract was harvested from a 1 L culture.

### Molecular and structural characterization

Protein samples were analyzed by SDS-PAGE. The protein band corresponding to the NiDODA enzyme had an estimated molecular weight of 35 kDa (Fig. 2A), consistent with the molecular weight calculated based on the protein sequence (33.41 kDa). Determination of the exact molecular mass was carried out through HPLC-ESI-Q-TOF-MS analysis using pure NiDODA protein. The mass spectrum obtained showed a peak with a mass of 33.324 kDa, similar to the weight estimated by SDS-PAGE and to the theoretical mass calculated.

The dioxygenase enzyme from *N. irregularis* was digested with trypsin to determine its peptide mass fingerprint through HPLC-MS/MS analysis. The main peptides identified corresponded to masses 927.46 *m/z* (AFDNALYEAITSPQSAR), 758.06 *m/z* (LVFNQIQPGLGWAMYYYFKP), 812.74 *m/z* (SHPTGEHLMPIYVALGAAGSDNAK), 596.83 *m/z* (GLDHGAWVPLK), and 1114.87 *m/z* (QPVYFLSHGGPSSMLEIESAPYQYWTAVGK). The pure protein was also analyzed by gel filtration chromatography to determine its quaternary structure, whether it is a monomer or could form oligomers. Different protein concentrations were tested, obtaining in all analyses a single peak with an estimated mass of 20.2 kDa (Fig. 2B). Despite the lower molecular mass obtained by gel filtration, it did not exceed the precise value obtained by HPLC-ESI-TOF-MS (33.324 kDa), thus the results showed that the 4,5-DODA protein from *N. irregularis* was a monomer.

The 3D modeling of the NiDODA was initially performed using the comparative modeling engine ProMod3 (Biasini et al. 2014; Bienert et al. 2017). This model was later compared with the only known crystallized protein capable of forming betalamic acid in enzymatic assays, the YgiD protein (*E. coli*, PDB accession number 2PW6) (Gandía-Herrero and García-Carmona 2014). Bioinformatic techniques predicted a homo-dimeric protein structure (Suppl. material 1: fig. S3). However, using the novel protein structure prediction tool AlphaFold (Jumper et al. 2021; Varadi et al. 2022), the NiDODA enzyme was modelled as a monomer (Fig. 2C), supporting the results obtained experimentally. Sequence comparison between the NiDODA and *E. coli* YgiD revealed a 25.20% identity. The three histidine residues involved in metal coordination in *E. coli* YgiD (His22, His57, and His234), which are strictly conserved, are also present in the NiDODA protein as His10, His48, and His230 (Gandía-Herrero and García-Carmona 2014). Structural comparison between both proteins was carried out with the alignment algorithm TM-align in the RCSB pairwise structure alignment tool (Bittrich et al. 2024). Overall structural comparison is shown in Fig. 2D, and a dynamic representation is provided in Suppl. material 3. A close-up view of the three conserved histidine residues involved in the configuration of the active site of the enzyme is shown in Fig. 2E, with a comparison between the positions in the novel enzyme with that from *E. coli*. A full sequence alignment incorporating structural considerations was performed using Expresso (Armougom et al. 2006) and visualized with ESPript (Robert and Gouet 2014), as shown in Fig. 2F.

### Kinetic characterization

ICP-MS studies were conducted to determine the potential cofactors used by the enzyme during catalysis. ICP analysis results showed that the purified protein contained 0.07%, 0.09%, 1.59%, 1.58%, and 0.01% of Zn, Cu, Fe, Co, and Ni, respectively. Considering that the detected Co levels were mainly due to the chromatographic column used (HIS-Select Cobalt Affinity Gel), the results indicated Fe as the enzyme’s potential cofactor. Thus, to maintain enzymatic activity, Fe was added to the purification buffers. Ultimately, Fe-supplemented purification increased the metal content in the purified sample by up to 8.5 times compared to the unsupplemented sample.

The kinetic characterization of NiDODA was performed spectrophotometrically by adding the purified enzyme to a reaction medium containing L-DOPA as substrate. The yellow coloration with a λ_max_ of 414 nm, which was only in the medium containing the enzyme, evidenced the production of betalamic acid, confirming that the purified enzyme has 4,5 extradiol-dioxygenase activity. The colorimetric properties of the betalamic acid obtained in the reaction match with the properties reported for betalamic acid produced by enzymes of plants and fungi (Trezzini et al. 1991; Mueller et al. 1997).

**Figure 3.**
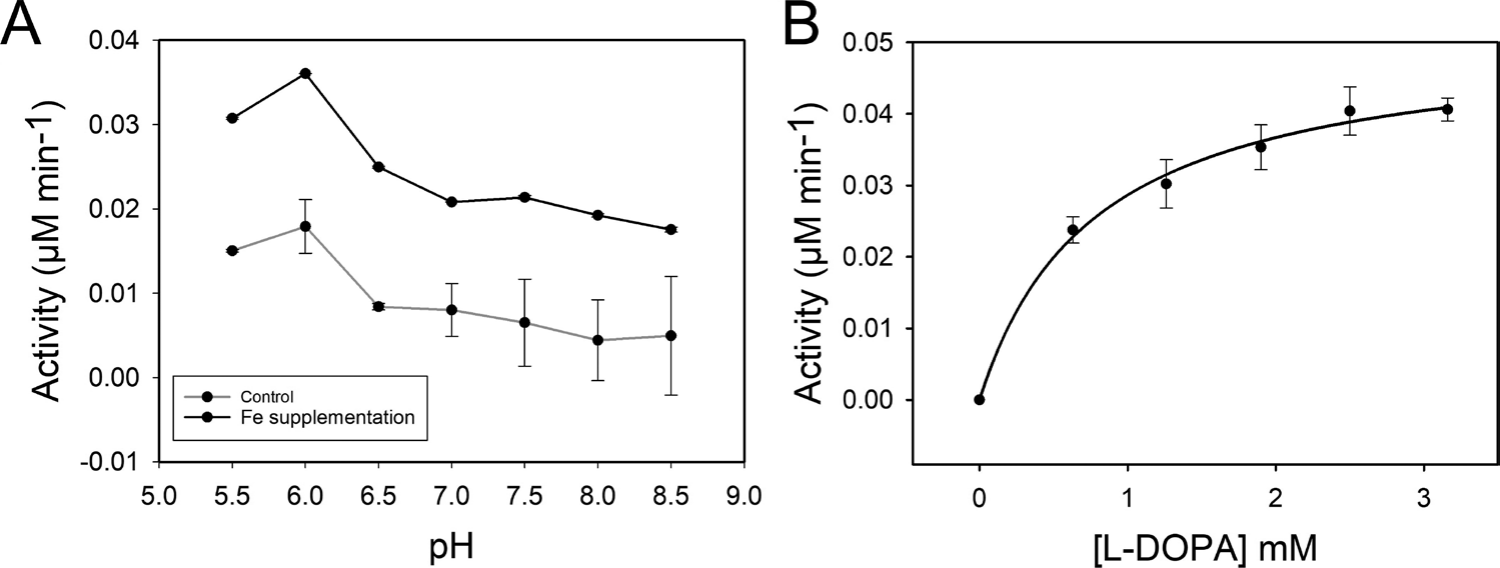
Characterization of the enzymatic activity of the NiDODA. **A**. Effect of pH on the enzymatic activity using 2.5 mM L-DOPA as substrate. Reactions were performed in 50 mM phosphate buffer for pH values ranging from 5.5 to 8.5. **B**. Enzymatic activity at different L-DOPA concentrations, measured in a reaction medium with 50 mM sodium phosphate buffer, pH 6.0. All the measurements were followed at λ = 414 nm.

The pH dependence of enzymatic activity was studied using the purified enzyme both with and without Fe supplementation in the purification buffers. The results showed higher enzymatic activity values when the enzyme was purified with Fe-supplemented buffers compared to the control without supplementation (Fig. 3A). This suggests that the metal may act as a cofactor for the enzyme since its presence throughout the successive purification steps led to higher final enzymatic activity compared to the enzyme purified without supplementation. These findings align with previous ICP-MS experiments, where the higher Fe content compared to other metals in the pure protein sample indicated its potential role as an enzymatic cofactor. Most probably the metal ion is coordinated by the three histidine residues identified in the sequence and structure comparisons (His10, His48, and His230) (Fig. 2). These histidines are strictly conserved in betalamic acid-forming dioxygenases and coordinate the metal ion in the only experimental crystal structure elucidated (Gandía-Herrero and García-Carmona 2014). The optimal pH for maximum enzymatic activity was determined to be pH 6.0 using L-DOPA as the substrate (Fig. 3A).

Then, NiDODA reaction kinetics on L-DOPA substrate was analyzed. Measurements were performed at the previously determined optimal pH (pH 6.0). The results showed an increase in activity with higher L-DOPA concentrations (Fig. 3B). Fitting the steady-state velocities to the Michaelis-Menten equation yielded a Km value of 0.8 ± 0.1 mM and a V_max_ of 0.05 μM min^–1^.

### Functional characterization

**Figure 4.**
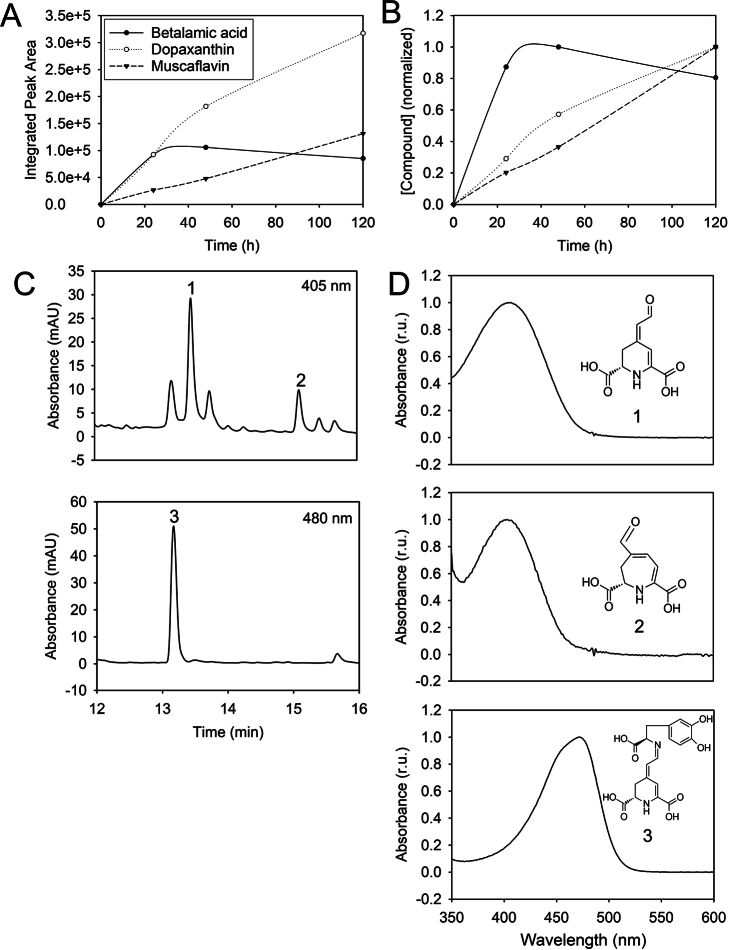
Time evolution of the enzymatic reaction in *E. coli* (pET-28a-NiDODA) cultures supplemented with L-DOPA. **A**. Time-course enzymatic reaction in *E. coli* (pET-28a-NiDODA) cultures measured by HPLC analysis, betalamic acid and muscaflavin were detected at λ = 405 nm, and dopaxanthin was detected at λ = 480 nm. **B**. Normalized data of panel **A**. **C**. HPLC chromatogram obtained at λ = 405 and 480 nm. Molecules were identified as betalamic acid (peak 1), muscaflavin (peak 2) and dopaxanthin (peak 3). **D**. Normalized spectra of compounds showed in panel **C**. Structures are shown inset. r.u., relative units.

The functional study of the novel NiDODA was conducted by detecting and monitoring the reaction intermediates of the betalain biosynthetic pathway. The results showed a rapid production of betalamic acid in the medium during the first 24 h, reaching its maximum concentration at 48 h (Fig. 4A, B). This compound, obtained from 4,5-DOPA-extradiol-dioxygenase activity on L-DOPA, was detected at a λ_max_ of 405 nm with a Rt of 13.5 min (Fig. 4C, D). Additionally, a peak at λ_max_ of 403 nm with a Rt of 15.1 min was also detected, corresponding to muscaflavin, a molecule produced by 2,3-DOPA-extradiol-dioxygenase activity.

The betalain derived from the condensation of betalamic acid with L-DOPA, dopaxanthin, was detected at a λ_max_ of 471 nm with a Rt of 13.1 min. Dopaxanthin progressively accumulated over 120 h, while the amount of betalamic acid decreased due to its condensation with L-DOPA and the formation of the above-mentioned betaxanthin. The chromatograms at 405 nm and 480 nm of samples taken after 48 h, as well as the absorbance spectra of the three compounds involved in the betalain biosynthetic pathway, are shown in Fig. 4.

For the unequivocal identification of the detected metabolites, HPLC-ESI-TOF-MS analyses were performed. The exact masses determined for the reaction products catalyzed by the NiDODA enzyme showed protonated molecular ions [M+H]^+^ for the metabolites, with experimental exact masses of 391.1146, 212.0545, and 212.0547 *m/z*. The obtained values are consistent with the theoretically calculated masses for the L-DOPA-derived compounds: dopaxanthin (391.1136 *m/z*), betalamic acid, and muscaflavin (both 212.0553 *m/z*).

**Figure 5.**
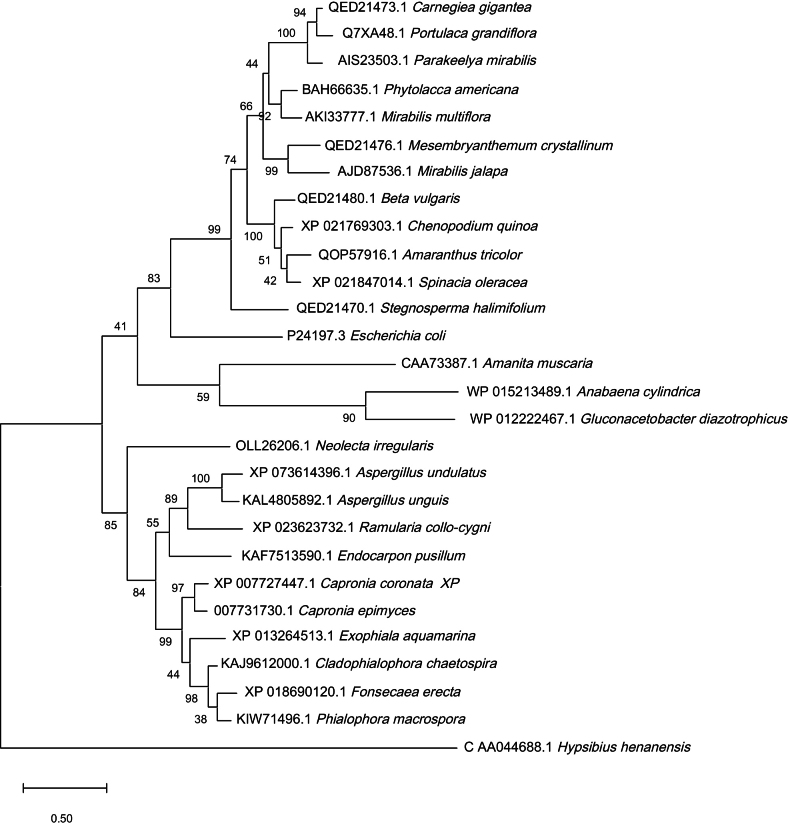
Phylogenetic tree of betalamic acid- producing DODA homologues. The numbers near the branches represent the percentage of replicate trees in which the associated sequences clustered together (1,000 replicates).

The functional analysis of NiDODA as a betalamic acid-producing enzyme showed that for the first time this dual activity has been characterized in an *Ascomycota* fungus. By using NiDODA as sequence reference, homologue sequences within *Ascomycota* were found in the NCBI database using the BLAST tool. The inferred sequences are mainly annotated as hypothetical or uncharacterized proteins. The top 10 hits, which showed a percent similarity above 40% to NiDODA, were included in a multiple sequences alignment alongside previously described betalain-producing enzymes in order to investigate their possible relationship with the betalain-producing capacity. As shown in Fig. 5, *Ascomycota* fungi sequences appeared clustered together without interlarding among other sequences. Similar results were obtained for *Caryophyllales* plant DODA enzymes which appeared clustered together. Interestingly, the only enzyme functionally described from an animal (*H. henanensis*) showed a lineage that branched off prior to the radiation of the ingroup made up of plants, fungi and microorganisms, and considered in this sense as an outgroup sequence. As observed, the *Ascomycota* sequences differ, with the *N. irregularis* sequence representing a novelty in this regard, while the previously known *A. muscaria* sequence is closer to those described in bacteria.

## Discussion

The yellow organism *N. irregularis* presents in its genome and transcriptome the sequence corresponding to an enzyme 4,5-DODA, key in the biosynthetic process of the yellow pigments betaxanthins, produced mainly by plants of the order *Caryophyllales* and only in some fungi. Thus, this species was screened for uncharacterized betalain-forming capacity. *N. irregularis* retains traits considered archaic, both morphologically and genetically, placing it in an intermediate position between filamentous fungi and yeasts. Specimens are scarce, so very little is known about the species. The pigment profile showed that the main pigment is riboflavin and betaxanthins were not detected (Fig. 1). Riboflavin is a water-soluble yellow pigment mainly produced by fungi and microorganisms such as *Ashbya gossypii*, *Candida famata*, and *Bacillus subtilis*, and its production is of great biotechnological interest since it is used as a food colorant (E-101) (Afroz Toma et al. 2023). Additionally, two more hydrophilic compounds were detected (Suppl. material 1: fig. S1A), probably corresponding to riboflavin degradation compounds based on similarities in the mass fragmentation pattern. The absorbance spectrum of riboflavin and all mass spectra are shown in Suppl. material 1: fig. S1B, C. The detection of riboflavin is relevant due to the basal situation of the genus *Neolecta* in the evolutionary history of *Ascomycota* (Spatafora et al. 2017) and the hypothesis that the earliest *Ascomycota* species were yeast-like fungi (Landvik 1996). Other species within the Phylum, like *A. gossypii*, *C. famata*, produce high quantities of this pigment to protect their spores from the UV radiation, among other functions, such as FAD and FMN synthesis or redox defense. The expression of riboflavin synthase and riboflavin kinase and the subsequent key enzymes in riboflavin metabolism pathway was detected in this work as supported by the RNA-seq data deposited in GEO with the accession number GSE300630. The results showed that 24 genes related to riboflavin metabolism were present in the samples, suggesting that the pigment plays a relevant role in the physiology of the fungus.

Although no betalains were detected in the fungus, RNA-seq analysis demonstrated that the key enzyme in betalain formation (4,5-DODA) is expressed in the organism. The gene corresponding to the 4,5-DODA enzyme (protein ID 2980) is expressed at the same level in the two independent samples analyzed. Therefore, the RNA-seq results suggest that while the fungus possesses the genetic framework for the betalain biosynthetic pathway (Suppl. material 2), it is unable to synthesize these pigments due to a lack of *de novo* L-tyrosine production. Consequently, the transcriptomic profile confirms why the betalain pathway remains incomplete despite the presence of key downstream enzymes.

Since it cannot be cultivated due to its possible symbiotic nature with plants (Landvik et al. 2003), the next step was to perform a heterologous expression of NiDODA in *E. coli* cells, which allowed us to analyze *in vitro* the betalain-forming capacity (Nguyen et al. 2017; Contreras-Llano et al. 2019). The sequence OLL26206.1 was used to construct an expression vector to produce the putative enzyme in *E. coli* cells. Molecular and structural characterization of the produced enzyme showed that the 4,5-DODA protein from *N. irregularis* was a monomer. Therefore, it is the first monomeric fungal enzyme producing betalamic acid described to date. The obtained enzyme was able to produce betalamic acid and dopaxanthin, an indication of the 4,5-DOPA-extradiol-dioxygenase activity. Other enzymes with such activity, include those from the fungus *A. muscaria* (Girod and Zryd 1991); the plants *M. jalapa* (Sasaki et al. 2009), *B. vulgaris* (Gandía-Herrero and García-Carmona 2012; Guerrero-Rubio et al. 2023), and *C. quinoa* (Henarejos-Escudero et al. 2022); the bacterium *G. diazotrophicus* (Contreras-Llano et al. 2019); and the cyanobacterium *A. cylindrica* (Guerrero-Rubio et al. 2020). Within the fungal species, the ability to synthesize betalains has only been described in the genera *Amanita*, *Hygrocybe*, and *Hygrophorus* (Babos et al. 2011). The obtained results add *Neolecta* as the fourth fungal genus described as capable of producing these pigments.

The kinetic characterization of the enzyme showed an optimal pH of 6.0 using L-DOPA as the substrate (Fig. 3A). The optimal pH value obtained contrasts with those reported for other DODA enzymes: 8.5 for the DODAs of *A. muscaria* (Girod and Zryd 1991) and *B. vulgaris* (Gandía-Herrero and García-Carmona 2012); 8 for the YgiD protein from *E. coli* (Gandía-Herrero and García-Carmona 2014); and 7, 6.5, and 5.5 for the DODAs from *A. cylindrica* (Guerrero-Rubio et al. 2020), *G. diazotrophicus* (Contreras-Llano et al. 2019), and *C. quinoa* (Henarejos-Escudero et al. 2022), respectively. The 4,5-DODA from *A. muscaria*, has a Km of 4.2 mM using L-DOPA as substrate, a Km value 5-fold higher than that of NiDODA (Soares et al. 2022). In plants, DODA enzymes from *B. vulgaris* and *C. quinoa* showed Km values of 6.9 and 1.5 mM, respectively, for the same substrate (Gandía-Herrero and García-Carmona 2012; Henarejos-Escudero et al. 2022). In the bacteria, betalain-producing dioxygenases described in *E. coli*, *G. diazotrophicus*, and *A. cylindrica* exhibited Km values of 7.9, 1.36, and 53,000 mM, respectively (Gandía-Herrero and García-Carmona 2014; Contreras-Llano et al. 2019; Guerrero-Rubio et al. 2020). Moreover, the enzyme from the aquatic organism *A. cylindrica* displayed substrate inhibition (Guerrero-Rubio et al. 2020). Therefore, NiDODA is the enzyme with the highest affinity towards L-DOPA substrate of those described in the literature, and consequently, the most efficient at binding and converting the substrate into product.

The study of the reaction intermediates detected by HPLC-ESI-TOF-MS showed that the NiDODA was able to produce muscaflavin, betalamic acid, and dopaxanthin. Thus, the NiDODA exhibits dual 2,3- and 4,5-DOPA-extradiol-dioxygenase activity, similar to the dioxygenase enzyme isolated from the fungi *A. muscaria* (Girod and Zryd 1991), the bacterium *G. diazotrophicus* (Contreras-Llano et al. 2019), and the cyanobacterium *A. cylindrica* (Guerrero-Rubio et al. 2020) whereas plant betalain-producing DODAs are specialized in the 4,5-cleavage of L-DOPA. Taking this into account, it was plausible to expect NiDODA to be closer, in terms of sequence evolution, to those betalain-producing enzymes with dual activity than those with a single activity. The performed phylogenetic tree (Fig. 5) shows a compact clade formed by plant sequences, separate from those of fungi and bacteria. Similarly, NiDODA appeared in a separate branch that comprises *Ascomycota* sequences, confirming the evolutionary distance to plant dioxygenases. Having extended this phylogenetic tree to include *Ascomycota* sequences obtained after BLAST search by using NiDODA as template, it shows that NiDODA might not be the only *Ascomycota* species with an enzyme capable of producing the catalytic opening of the L-DOPA ring to produce betalains. Also in this sense, the multiple sequence alignment of *Ascomycota* proteins (Suppl. material 1: fig. S4) shows that the three highly conserved histidine -detected in NiDODA as His10, His48 and His230- related to metal coordination in DODA enzymes are also present in these uncharacterized proteins. Thus, the obtained results open the possibility of these fungal proteins being studied as putative betalain-producing enzymes with a similar activity to the one here demonstrated for NiDODA.

After decades of studies with DODA enzymes from plants, the current information about proteins with betalain-producing potential, found in fungi, bacteria and animals, seems to indicate their widespread presence in nature, which makes it more challenging to draw conclusions about a single evolutionary route from a common ancestor. In fact, it has been proven that L-DOPA dioxygenase enzymes in plants do not share a common ancestor, but their activity in *Caryophyllales* plant species comes from different events of gene duplication and neofunctionalization. This phenomenon has been described to happen at least in three independent origins (Walker‐Hale et al. 2025) where lineage-specific mutations finally converge in the obtention of enzymes specialized in the production of betalains by the metabolization of L-DOPA. The extended phylogenetic tree here inferred, and the experimental characterization of the betalain-forming activity of NiDODA support the idea of a convergent evolution of DODA enzymes not only in plants but also extended to other organisms. Thus, the well-defined group where *Ascomycota* species are clustered together might indicate an independent radiation of betalain-producing enzymes, but further studies would be needed in this direction. In *Caryophyllales* plants, betalains play important roles in photoprotection, improve abiotic stress resistance, and attract pollinators for seed dispersal (Jain and Gould 2015; Marshall and Johnsen 2017). However, the biological significance of betalain-producing enzymes in bacteria and fungi remains unknown and the presence of these enzymes in such diverse organisms might also derive from independent evolutionary strategies that have converged in the obtention of efficient L-DOPA dioxygenases, with a similar activity to the betalain-producing enzymes of plants (Guerrero-Rubio et al. 2025). The occurrence of the same catalytic reaction in enzymes with a considerable disparity of sequences is something common in nature (Riziotis et al. 2025) and not exclusive for the production of betalains. The structural similarity described in this work is noteworthy (Fig. 2) despite the sequence distance, and further analyses are needed to understand the extended appearance of L-DOPA dioxygenase enzymes, in addition to considering a possible unknown function for these enzymes beyond the production of betalains, especially in species that do not belong to *Plantae*.

## Conclusions

In conclusion, the key enzyme in the betalain biosynthetic pathway, 4,5-DOPA-extradiol-dioxygenase (DODA), is present in the primitive organism *N. irregularis*. Although betalains have not been described in the native conditions for this species, its pigments were characterized for the first time, with riboflavin identified as the pigment responsible for the yellow coloration and fluorescence of its fruiting bodies. The first transcriptomic analysis of the species shows gene expression of the NiDODA. The enzyme sequence from *N. irregularis* was optimized for recombinant expression in *E. coli*, purified, and characterized for the first time as a monomeric protein of 33.4 kDa. When L-DOPA was used as substrate, the enzymatic activity resulted in the formation of betalamic acid and muscaflavin, due to its dual 2,3- and 4,5-DOPA extradiol-dioxygenase activity, and it turned out to be the most efficient enzyme at binding the substrate in the formation of betalains. This study fully describes the second fungal enzyme capable of producing betalains and broadens the known phylogenetic distribution of betalain biosynthesis to include highly distinctive and evolutionarily unique non-plant organisms. These findings provide a new insight into the evolutionary breadth of DOPA-dioxygenases outside *Plantae* showing that key metabolic innovations associated with betalains may have deeper and more ancient origins than previously appreciated.

## Additional information

### Artificial Intelligence (AI) use

The authors accept full responsibility for the content of the manuscript, including the disclosure of any use of AI.

No AI tools were used in the preparation of this manuscript.

### Adherence to national and international regulations

All the fungal strains used in this study have been legally obtained, respecting the Convention on Biological Diversity (Rio Convention).

### Author ORCIDs

P. Martínez-Rodríguez https://orcid.org/0000-0003-1682-5536

M.A. Guerrero-Rubio https://orcid.org/0000-0002-3261-2058

P. Henarejos-Escudero https://orcid.org/0000-0003-4295-2459

S. Hernández-García https://orcid.org/0000-0002-6944-0050

F. Gandía-Herrero https://orcid.org/0000-0003-4389-3454

### Data availability

The RNA-Seq data discussed in this publication have been deposited in NCBI's Gene Expression Omnibus (Edgar et al. 2002) and are accessible through GEO Series accession number GSE300630 (https://www.ncbi.nlm.nih.gov/geo/query/acc.cgi?acc=GSE300630). Fasta sequences, alignments and phylogenetic tree used in the manuscript are available at Zenodo database at https://zenodo.org/records/17191542?token=eyJhbGciOiJIUzUxMiJ9.eyJpZCI6IjZjZTA1MjdhLTY3N2UtNDBhZC05NTM0LTU4YjUxMWE4MWRjOCIsImRhdGEiOnt9LCJyYW5kb20iOiI4MmUzM2RhZWZiNmY4YTFiOGU3NGUxZGY3MjA0YjVlMyJ9._J5SYbhw4p5GouaxqLDHcDofDI82PY1mjt3ppfo-47R7PkDNJwTCqhFoIGcCXqwYbZcpiXGvKWqPpZ96ZZsOIQ. The rest of the datasets generated during and/or analyzed during the current study are available from the corresponding author on reasonable request.

## Supplementary materials

Supplementary material 1 Supplementary information Data type docx Explanation note **figure S1**. Determination of main pigments from the *N. irregularis* extract. **figure S2**. Optimization of induction conditions for NiDODA expression in *E. coli* cells. **figure S3**. 3D homodimer of the NiDODA. **figure S4**. CLUSTAL Omega multiple alignment of *Ascomycota* fungi sequences. **table SS1**. DODA enzymes used for phylogenetic analysis. **table S2**. Summary of the sequencing data generated for RNA-seq and mapping of the *N. irregularis* genome. https://binary.pensoft.net/file/1670851This dataset is made available under the Open Database License (http://opendatacommons.org/licenses/odbl/1.0/). The Open Database License (ODbL) is a license agreement intended to allow users to freely share, modify, and use this Dataset while maintaining this same freedom for others, provided that the original source and author(s) are credited. Pedro Martínez-Rodríguez, M. Alejandra Guerrero-Rubio, Paula Henarejos-Escudero, Samanta Hernández-García, Fernando Gandía-Herrero

Supplementary material 2 RNA-seq annotation of *Neolecta irregularis* Data type xlsx https://binary.pensoft.net/file/1670852This dataset is made available under the Open Database License (http://opendatacommons.org/licenses/odbl/1.0/). The Open Database License (ODbL) is a license agreement intended to allow users to freely share, modify, and use this Dataset while maintaining this same freedom for others, provided that the original source and author(s) are credited. Pedro Martínez-Rodríguez, M. Alejandra Guerrero-Rubio, Paula Henarejos-Escudero, Samanta Hernández-García, Fernando Gandía-Herrero

Supplementary material 3 Dynamic comparison of the structure for the NiDODA with the YgiD protein from *Escherichia coli* Data type mp4 https://binary.pensoft.net/file/1670853This dataset is made available under the Open Database License (http://opendatacommons.org/licenses/odbl/1.0/). The Open Database License (ODbL) is a license agreement intended to allow users to freely share, modify, and use this Dataset while maintaining this same freedom for others, provided that the original source and author(s) are credited. Pedro Martínez-Rodríguez, M. Alejandra Guerrero-Rubio, Paula Henarejos-Escudero, Samanta Hernández-García, Fernando Gandía-Herrero
