# Supplementary material for: A functional betalain-producing dioxygenase in Neolecta irregularis reveals an expanded evolutionary origin of the pathway
Source: IMA Fungus. 2026 Jun 5;17:e184549. doi: 10.3897/imafungus.17.184549 (PMC13263728; doi:10.3897/imafungus.17.184549)
Supplement: Supplementary material 1 — Supplementary information [file imafungus-17-e184549-s001.docx]

**A Functional Betalain‑Producing Dioxygenase in Neolecta irregularis Reveals an Expanded Evolutionary Origin of the Pathway**

Martínez-Rodríguez P^a, *^, Guerrero-Rubio M.A^a, b, *^, Henarejos-Escudero P^a, 1^, Hernández-García S^a⸸^, Gandía-Herrero F^a^

^a^ Departamento de Bioquímica y Biología Molecular A, Unidad Docente de Biología, Facultad de Veterinaria. Regional Campus of International Excellence "Campus Mare Nostrum", Universidad de Murcia, Campus de Espinardo, 30100, Murcia, Spain.

^b^Faculty of Biomedical and Health Sciences, Universidad Europea de Valencia, Valencia, Spain.

^1^ Current Address: Molecular Recognition and Encapsulation Research Group (REM), Health Sciences Department, Universidad Católica de Murcia (UCAM), Campus de los Jerónimos 135, 30107 Guadalupe, Spain.

* The authors have contributed equally to the manuscript

^⸸^ Corresponding author (Tel: +34 868 884786; E-mail: samanta.hernandez@um.es)

Emails:

Pedro Martínez-Rodríguez: [pedro.martinezr@um.es](mailto:pedro.martinezr@um.es)

ORCID: [0000-0003-1682-5536](https://orcid.org/0000-0003-1682-5536)

M. Alejandra Guerrero-Rubio: [mariaalejandra.guerrero@universidadeuropea.es,](mailto:mariaalejandra.guerrero@universidadeuropea.es) [mariaalejandra.guerrero@um.es](mailto:mariaalejandra.guerrero@um.es)

ORCID: [0000-0002-3261-2058](https://orcid.org/0000-0002-3261-2058)

Paula Henarejos-Escudero: [paula.henarejos@um.es](mailto:paula.henarejos@um.es); phenarejos@ucam.edu

ORCID: [0000-0003-4295-2459](https://orcid.org/0000-0003-4295-2459)

Samanta Hernández-García: [samanta.hernandez@um.es](mailto:samanta.hernandez@um.es)

ORCID: [0000-0002-6944-0050](https://orcid.org/0000-0002-6944-0050)

Fernando Gandía-Herrero: [fgandia@um.es](mailto:fgandia@um.es)

ORCID: [0000-0003-4389-3454](https://orcid.org/0000-0003-4389-3454)

**SUPPLEMENTARY MATERIAL**

**
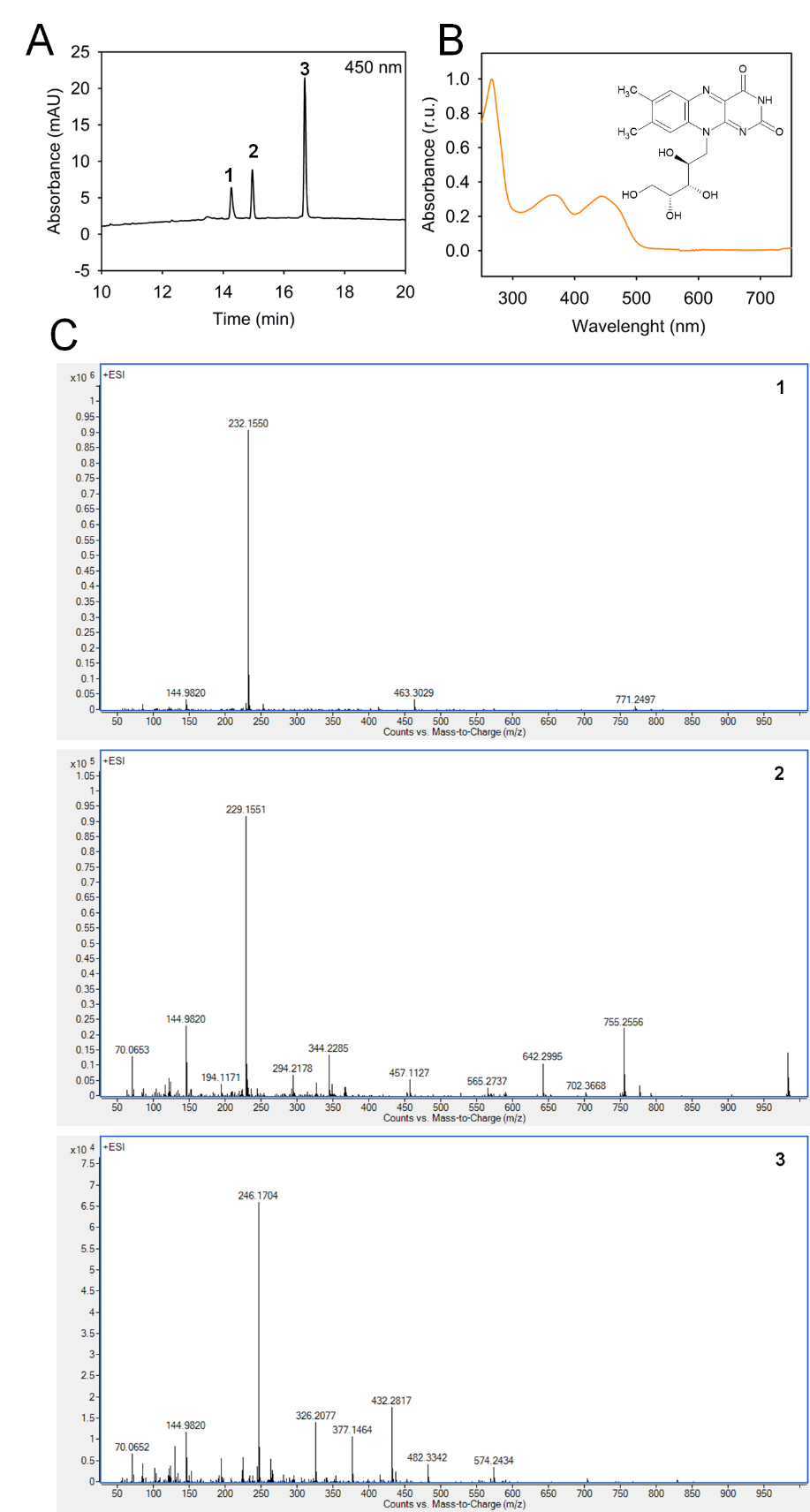
**

**Supplementary Figure S1.** HPLC chromatogram obtained at λ = 450 nm from the *N. irregularis* extract (A), showing two unidentified compounds (peaks 1 and 2) and riboflavin (peak 3). (B) Normalized absorbance spectrum of riboflavin (peak 3). (C) Mass spectra of the compounds shown in panel A. AU: Absorbance Units; r.u.: relative units.


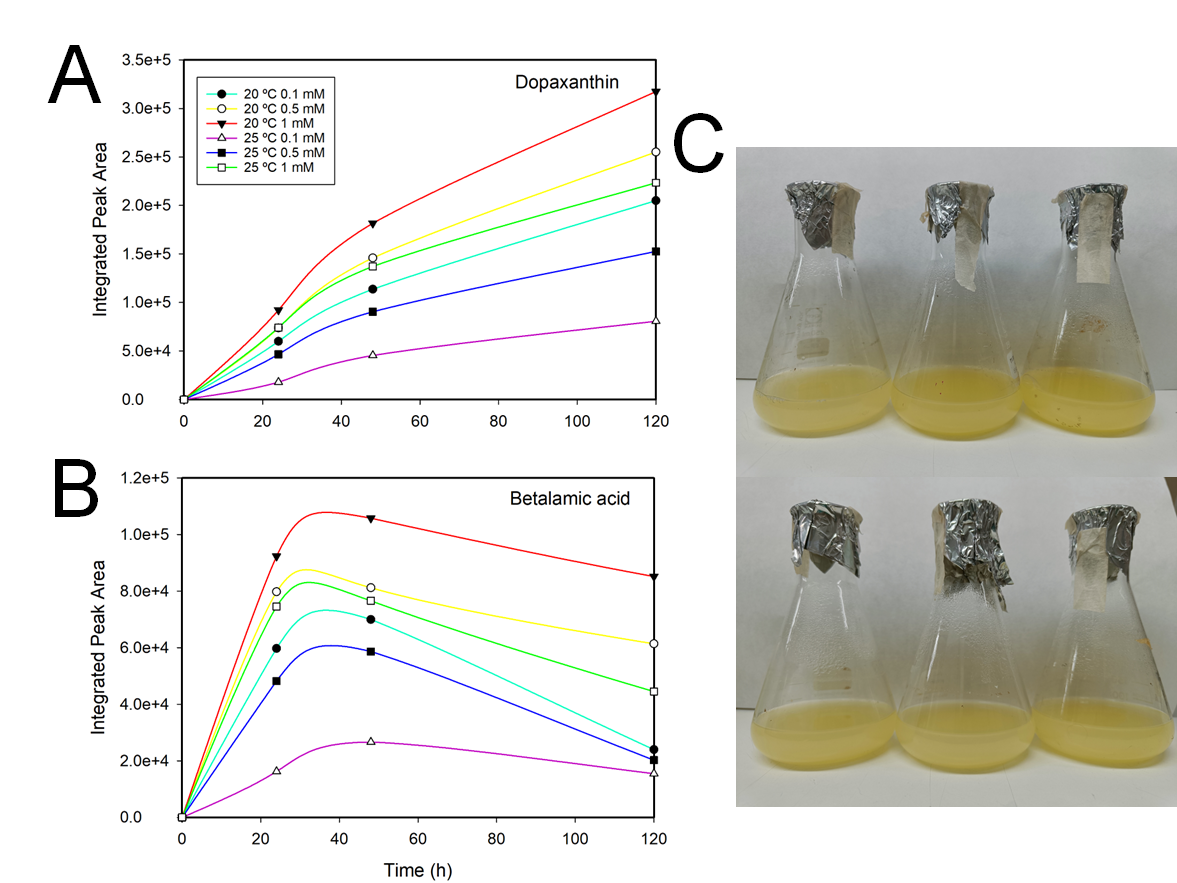


**Supplementary Figure S2**. Optimization of induction conditions for NiDODA expression in *E. coli* cells. HPLC monitoring of compounds derived from dopaxanthin (A) and betalamic acid (B) enzymatic activity is shown; as well as macroscopic images of *E. coli* solutions (pET-28a-NiDODA) after induction with 0.1 (left), 0.5 (center) and 1 (right) mM IPTG at 20 (top) or 25 (bottom) ºC after 24 h incubation with L-DOPA.


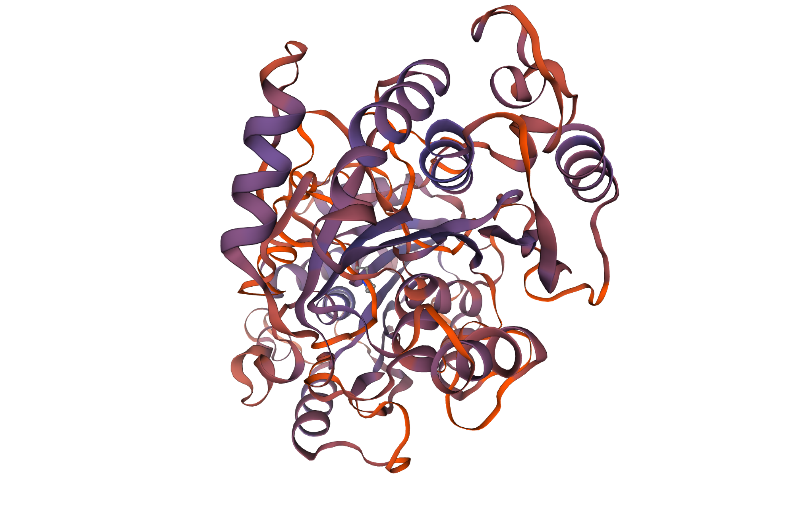


**Supplementary Figure S3.** 3D homodimer of the NiDODA obtained using the comparative modelling engine ProMod3.

OLL26206.1_Neolecta_irregularis ------------------------------------------------------------ 0

XP_023623732.1_Ramularia_collo-cygni ------------------------------------------------------------ 0

XP_073614396.1_Aspergillus_undulatus MPSTTNEQRKTE--RPSITAISLVTLAVAIALALVFGSSEPANLFGLRRFAQASWAAIGS 58

KAL4805892.1_Aspergillus_unguis MSSTASKRPNTERNPPSITVISVITLAAGILIAVLVGSTNPANVFGLRRFAQSSWAAIKS 60

KAF7513590.1_Endocarpon_pusillum ----------------------MIDLAAFISRAQGIKTSLL-GATELSRYSLRSAQTKLS 37

XP_013264513.1_Exophiala_aquamarina ------------------------------------------------------------ 0

XP_007727447.1_Capronia_coronata ------------------------------------------------------------ 0

XP_007731730.1_Capronia_epimyces ------------------------------------------------------------ 0

XP_018690120.1_Fonsecaea_erecta ------------------------------------------------------------ 0

KAJ9612000.1_Cladophialophora_chaetospira ------------------------------------------------------------ 0

KIW71496.1_Phialophora_macrospora ---------MLRDIRAATATIASISALVILLLAVRLHSSSPFHQEDFDDLALSRLSTPLR 51

OLL26206.1_Neolecta_irregularis ----------------------------MRQPVYFLSHGGPSSMLEIESAPYQYWTAVGK 32

XP_023623732.1_Ramularia_collo-cygni --------------------------MTTRTPVYFLSHGGPNIMEDTKHPAYAKLQEIGH 34

XP_073614396.1_Aspergillus_undulatus --RRLVSSLAGVERAQDTPRTSAIDEFTMRTPVYFLSHGGPNIMYDVEHPAYKELGRIGR 116

KAL4805892.1_Aspergillus_unguis PAPRLVSTTAAQASLQTSP---AVYGSNMKTPVYFLSHGGPSVMYDTEHPAYKKLGEIGR 117

KAF7513590.1_Endocarpon_pusillum PQGRNPPH--------------TTTMTAQKTPIYFLSHGGPQTCHTPTHPVYPQLQSIGR 83

XP_013264513.1_Exophiala_aquamarina --------------------------MASKAPVYFVSHGGPNTMYDNKHPVWAELQAIGR 34

XP_007727447.1_Capronia_coronata --------------------------MAKRTPVYFVSHGGPNTMYETNHPVYPQLQRIGK 34

XP_007731730.1_Capronia_epimyces --------------------------MAKRTPVYFVSHGGPNTMYETSHPVYPQLQRIGK 34

XP_018690120.1_Fonsecaea_erecta --------------------------MAKRTPVYFVSHGGPNTMFETEHPVYPQLQRIGR 34

KAJ9612000.1_Cladophialophora_chaetospira --------------------------MAKRTPVYFVSHGGPNTMYETDHPVYPKLQSIGK 34

KIW71496.1_Phialophora_macrospora LLSNLVKPSS------------TNTTMAIRTPVYFVSHGGPNTMYEVNHPVYPQLQSIGK 99

: *:**:*****. : :*:

OLL26206.1_Neolecta_irregularis EIKS--SKPEGIVIFSAHWDS-DQEGEVDVSFDED-NELIYDFYGFPAHYYNVKFRSKNP 88

XP_023623732.1_Ramularia_collo-cygni EITG-KVKPKAIVVFSGHWQG--FQDTIEVNTMES-SPLIYDFYGFPAHYYEYKYPNKGS 90

XP_073614396.1_Aspergillus_undulatus EITT-QVKPRAVVVFSAHWQA--GRDTVQVNTAEI-TELIYDFYGFPSHYYEEKYPNVGS 172

KAL4805892.1_Aspergillus_unguis EITT-KVKPRAVVVFSAHWQA--GRDTVQVNTAEM-TELIYDFYGFPSHYYEEKYPNVGS 173

KAF7513590.1_Endocarpon_pusillum EISNTSTKPAAIVVFSAHWQSSSGPNTIEVNTSDKPLPLIYDFYGFPDHYYKTQFPYRAS 143

XP_013264513.1_Exophiala_aquamarina EITQ-KVKPSAIVVFSAHWQA-QRPNTIEVNISEQ-EPLLYDFYGFPRHYYAEKFPNKGS 91

XP_007727447.1_Capronia_coronata EITE-TVRPAAVVVFSAHWQA-DRPGTIEVNVAEE-EPLIYDFYGFPRHYYAEKFPNKGS 91

XP_007731730.1_Capronia_epimyces EITE-SVKPSAVVVFSAHWQA-DRPNTIEVNVAEE-EPLIYDFYGFPRHYYSEKFPNKGS 91

XP_018690120.1_Fonsecaea_erecta EITQ-QVKPAAIVVFSAHWQA-ARPNTIEVNVAED-EPLLYDFYGFPRNYYTEKFPNKGS 91

KAJ9612000.1_Cladophialophora_chaetospira EITT-QVKPSAIVVFSAHWQA-DRPNTIEVNVSED-EPLLYDYYGFPRHYYLEKFPNKGS 91

KIW71496.1_Phialophora_macrospora EITT-KVKPSAVVVFSAHWQA-ERPNTIEVNVAED-EPLLYDYYGFPRHYYMEKFPNKGS 156

**. :* .:*:**.**:. . ::*. : *:**:**** :** ::

OLL26206.1_Neolecta_irregularis SIFHNMIADTIHRSGFKTRGKKRGLDHGAWVPLKVMFDGAT---DIPICQVALPSRE-NP 144

XP_023623732.1_Ramularia_collo-cygni PELAEKILGMLQTAGIKAEGVRRGLDHGVWASFMCLFDPKTNPLDIPIVQVSLFDYF-KA 149

XP_073614396.1_Aspergillus_undulatus KEIANKVLDSFKQAGIKAEGVKRGLDHGVWASFKCAFEPDTNPLNVPVVQVSLFGTE-DP 231

KAL4805892.1_Aspergillus_unguis REIANKVLDSLNQAGIKAEGVKRGLDHGVWVGFKCAFEPESNPLNVPIVQVSLFNSE-DP 232

KAF7513590.1_Endocarpon_pusillum GKVSQRVMDVLNEGGVKAVGVERGLDHGVWVPFKVVFEGLEKEMP-PIVQVSLFGAE-DA 201

XP_013264513.1_Exophiala_aquamarina PELARKIMGLLNDNGVRTQEEERGLDHGAFVPFKVMFDLKENPVNVPIVQVSLFDDDTDA 151

XP_007727447.1_Capronia_coronata PEVAQKVMAALGENGIKTQGVKRGLDHGVFVPFKIMFDPATNPLTVPIVQVSLFDDDTDA 151

XP_007731730.1_Capronia_epimyces PKLAKQVMAILGENGIKTQEAERGLDHGVFVPFKVMFDPKTNPLTVPIVQVSLFDDDTDA 151

XP_018690120.1_Fonsecaea_erecta TKLAQHVIDVLAEHGIKTQKEERGLDHGVFVPFKVMFNPDTNPVNVPIVQVSLFDDHTDA 151

KAJ9612000.1_Cladophialophora_chaetospira AKIAQRVIDVLSDNGIRTEKVERGLDHGVFVPFKVMFNPEENPVKVPIVQVSLFDDETAA 151

KIW71496.1_Phialophora_macrospora AKVAQRVIDVLGENGIRTEKTERGLDHGVFVPFKVMFNPEKNPLTVPIVQVSLFNDDTDA 216

. . : : *.:: .******.:. : *: *: **:* .

OLL26206.1_Neolecta_irregularis EANIKIGKAASSLREENVLIICSGMAIHNLQVAMSDTPFEVDISPFK-AFDNALYEAIT- 202

XP_023623732.1_Ramularia_collo-cygni DLHFKLGKAIASLREEGVQIIVSGMAVHNLRDMGRMWG-QPGALDYTLSFDEALREAVEQ 208

XP_073614396.1_Aspergillus_undulatus MQHYRLGEAVSKLREENILIIVSGMAVHNLRDLRFTFG-DPRPMPYTASFDEALKDAATK 290

KAL4805892.1_Aspergillus_unguis MQHYRLGEAVAKLREENILIIVSGMAVHNLRDLRFTFR-DPRPLPYTASFDEALKDAATK 291

KAF7513590.1_Endocarpon_pusillum EEHLRLGRVVARLREENILIVVSGMAVHNLRDMWVTIR-DPKPLSYTASFDEALREAVES 260

XP_013264513.1_Exophiala_aquamarina EAHIKLGRAVQNLREENIVIICSGMSIHNLRHFMMGGG-RSGNLPGFANFDQALKDAAET 210

XP_007727447.1_Capronia_coronata AAHIRLGRAVQKLRDQNILVVASGMSVHNLRHMMMAMG-TGKTMPYSVTFDVALKEAVET 210

XP_007731730.1_Capronia_epimyces AAHIRLGRAVQKLRDENILIVASGMSVHNLRHMMTAMA-TGKTMPYSASFDAALKDAVET 210

XP_018690120.1_Fonsecaea_erecta AAHIKLGQAVQKLREENVLIICSGMSVHNLRHFMMLGGGGNRTMPYAVTFDEALKVAVET 211

KAJ9612000.1_Cladophialophora_chaetospira EAHIKLGRAVEKLREENILVICSGMSVHNLRHLMMLGRGSSKTMPYSITFDEALKVATET 211

KIW71496.1_Phialophora_macrospora AAHIKLGRAVEKLRDENILIICSGMSVHNLRHFMMLGRGGDRTMPYAVTFDEALKVAAET 276

: ::*.. **::.: :: ***::***: ** ** *

OLL26206.1_Neolecta_irregularis SPQSARETKMKKLMKHSEFRKSHPTGEHLMPIYVALGAAGSDNAKLVFNQIQPGLGWAMY 262

XP_023623732.1_Ramularia_collo-cygni DPS-SREEAMTQLLSRPDFKKAHPSADHLMPIYVGAGAASDEKATRIFTLPEGSMSWAQF 267

XP_073614396.1_Aspergillus_undulatus PPA-ERAQALTDLLKRGDARQAHPTFDHLLPIHVGAGAAGDDVGKRLWTLGEGSLSWAQY 349

KAL4805892.1_Aspergillus_unguis PPA-DRAQALTDLLKRGDARQAHPSFEHLLPIHIGAGAAGDDLGKRLWTLKEGSLSWAQY 350

KAF7513590.1_Endocarpon_pusillum DPGSDRDTRMKELVKRTDVKRAHPTLEHLLPIHVGVGAAGSDRGKRLWTMGEGSLSWAQF 320

XP_013264513.1_Exophiala_aquamarina KPGSHRDELMVKLLQRSDARLAHPTFEHLLPAHIAVGAAGEDKGKQLFTLVEGPLAWAQY 270

XP_007727447.1_Capronia_coronata KPGEARDEQMVKLLKRPDARQAHPTFEHLLPIHIAVGAAGEDPGKQLWTMADGSLAWAQY 270

XP_007731730.1_Capronia_epimyces EAGEARDEKMVKLLTRPDGRQAHPTFEHLLPIHIAVGAAGHDPGKQLWTLAEGSLAWAQY 270

XP_018690120.1_Fonsecaea_erecta TPGEQRDEAMIGLLERGDARLAHPTFEHLLPIHIAAGAAGTDQGKQLWTLQEASMGWAQY 271

KAJ9612000.1_Cladophialophora_chaetospira KPGEQRDENMIGLLERPDARQAHPTFEHLLPIHIAVGAAGGDQGKQTWTLQEGSMAWAQY 271

KIW71496.1_Phialophora_macrospora TPGEQRDENMIELLERPDARQAHPTFEHLLPIHIAAGAAGSDQGKQLWTLAEASMAWAQY 336

* : *: : : : :**: :**:* ::. ***. : .. :. : :.** :

OLL26206.1_Neolecta_irregularis YFKP-------------- 266

XP_023623732.1_Ramularia_collo-cygni RFGEVGAPAS-------- 277

XP_073614396.1_Aspergillus_undulatus RFGEVTNASNAV------ 361

KAL4805892.1_Aspergillus_unguis RFGDLGSVSAL------- 361

KAF7513590.1_Endocarpon_pusillum RFGEVRSG---------- 328

XP_013264513.1_Exophiala_aquamarina RFGEVAAAS--------- 279

XP_007727447.1_Capronia_coronata KFEAEEGTREEAQRL--- 285

XP_007731730.1_Capronia_epimyces RFEAGGDQVKEDQEAHHL 288

XP_018690120.1_Fonsecaea_erecta RFGEVGAS---------- 279

KAJ9612000.1_Cladophialophora_chaetospira RFGEVEA----------- 278

KIW71496.1_Phialophora_macrospora RFGELAA----------- 343

*

**Supplementary Figure S4. CLUSTAL Omega multiple alignment of *Ascomycota* fungi sequences.** The highly conserved histidine detected in betalain-producing enzymes are also detected in the analysed sequences (yellow highlight).

**Supplementary Table S1.** Protein Accession (NCBI/INSDC/RefSeq) corresponding to the 4,5-DOPA-extradiol-dioxygenase (DODA) enzymes, both characterized and putative, used in the phylogenetic analysis. References of characterized DODA enzymes are included.

| **Organism** | **Protein Accession (NCBI/INSDC/RefSeq)** | **Kingdom** | **Reference** |
| --- | --- | --- | --- |
| *Beta vulgaris* | QED21480.1 | Planta | Chang et al., 2021 |
| *Chenopodium quinoa* | XP_021769303.1 | Planta |  |
| *Mirabilis jalapa* | AJD87536.1 | Planta |  |
| *Portulaca grandiflora* | Q7XA48.1 | Planta |  |
| *Phytolacca americana* | BAH66635.1 | Planta |  |
| *Carnegiea gigantea* | QED21473.1 | Planta |  |
| *Mesembryanthemum crystallinum* | QED21476.1 | Planta |  |
| *Amaranthus tricolor* | QOP57916.1 | Planta |  |
| *Parakeelya mirabilis* | AIS23503.1 | Planta |  |
| *Stegnosperma halimifolium* | QED21470.1 | Planta |  |
| *Spinacia oleracea* | XP_021847014.1 | Planta |  |
| *Mirabilis multiflora* | AKI33777.1 | Planta |  |
| *Amanita muscaria* | CAA73387.1 | Fungi | Soares et al., 2022 |
| *Neolecta irregularis* | OLL26206.1 | Fungi |  |
| *Aspergillus undulatus* | XP_073614396.1 | Fungi |  |
| *Aspergillus unguis* | KAL4805892.1 | Fungi |  |
| *Capronia coronata* | XP_007727447.1 | Fungi |  |
| *Capronia epimyces* | XP_007731730.1 | Fungi |  |
| *Cladophialophora chaetospira* | KAJ9612000.1 | Fungi |  |
| *Endocarpon pusillum* | KAF7513590.1 | Fungi |  |
| *Exophiala aquamarina* | XP_013264513.1 | Fungi |  |
| *Fonsecaea erecta* | XP_018690120.1 | Fungi |  |
| *Ramularia collo-cygni* | XP_023623732.1 | Fungi |  |
| *Anabaena cylindrica* | WP_015213489.1 | Bacteria | Guerrero-Rubio et al., 2020 |
| *Escherichia coli* | P24197.3 | Bacteria | Gandía-Herrero & García-Carmona, 2014 |
| *Gluconacetobacter diazotrophicus* | WP_012222467.1 | Bacteria | Contreras-Llano et al., 2019 |
| *Hypsibius henanensis* | C_AA044688.1 * | Animal | Li et al., 2024 |

*Deposited in GenBase (CNCB)

**References:**

Chang, Y. C., Chiu, Y. C., Tsao, N. W., Chou, Y. L., Tan, C. M., Chiang, Y. H., ... & Yang, J. Y. (2021). Elucidation of the core betalain biosynthesis pathway in Amaranthus tricolor. Scientific Reports, 11(1), 6086.

Contreras-Llano, L. E., Guerrero-Rubio, M. A., Lozada-Ramírez, J. D., García-Carmona, F., & Gandía-Herrero, F. (2019). First betalain-producing bacteria break the exclusive presence of the pigments in the plant kingdom. MBio, 10(2), 10-1128.

Gandía-Herrero, F., & García-Carmona, F. (2014). Escherichia coli protein YgiD produces the structural unit of plant pigments betalains: characterization of a prokaryotic enzyme with DOPA-extradiol-dioxygenase activity. Applied Microbiology and Biotechnology, 98(3), 1165-1174.

Guerrero-Rubio MA, García-Carmona F, Gandía-Herrero F (2020) First description of betalains biosynthesis in an aquatic organism: characterization of 4,5-DOPA-extradiol-dioxygenase activity in the cyanobacteria Anabaena cylindrica. Microbial Biotechnology 13: 1948–1959.

Li L, Ge Z, Liu S, Zheng K, Li Y, Chen K, Fu Y, Lei X, Cui Z, Wang Y, Huang J, Liu Y, Duan M, Sun Z, Chen J, Li L, Shen P, Wang G, Chen J, Li R, Li C, Yang Z, Ning Y, Luo A, Chen B, Seim I, Liu X, Wang F, Yao Y, Guo F, Yang M, Liu CH, Fan G, Wang L, Yang D, Zhang L (2024) Multi-omics landscape and molecular basis of radiation tolerance in a tardigrade. Science 386.

Soares DMM, Goncalves LCP, MacHado CO, Esteves LC, Stevani C V., Oliveira CC, Dörr FA, Pinto E, Adachi FMM, Hotta CT, Bastos EL (2022). Reannotation of Fly Amanita L-DOPA Dioxygenase gene enables Its Cloning and Heterologous Expression. ACS Omega 7: 16070–16079.

**Supplementary Table S2.** Summary of the sequencing data generated for RNA-seq and mapping of the *N. irregularis* genome.

| **Sample** | **Raw Reads** | **Clean Reads** | **GC Content**  **(%)** | **% ≥**  **Q30** | **Mapped Reads**  **(%)** |
| --- | --- | --- | --- | --- | --- |
| **31510** | 22294961 | 20968936 | 43.01 | 88.88 | 46.26 |
| **33461** | 28670032 | 25873189 | 44.10 | 88.86 | 46.73 |

**Supplementary Video 1.** The video shows a dynamic comparison of the structures for the NiDODA, characterized in this work and shown in blue, with the YgiD protein from *Escherichia coli*, shown in brown color. It is a mp4 file and lasts for 7.5 seconds.
